# Tempering Improves Flour Properties of Refined Intermediate Wheatgrass (*Thinopyrum intermedium*)

**DOI:** 10.3390/foods8080337

**Published:** 2019-08-10

**Authors:** Catrin Tyl, Radhika Bharathi, Tonya Schoenfuss, George Amponsah Annor

**Affiliations:** Department of Food Science and Nutrition, University of Minnesota, 1334 Eckles Avenue, Saint Paul, MN 55108, USA

**Keywords:** flour refinement, intermediate wheatgrass, sustainability, tempering, bran

## Abstract

Progress in breeding of intermediate wheatgrass (*Thinopyrum intermedium*), a perennial grain with environmental benefits, has enabled bran removal. Thus, determination of optimum milling conditions for production of refined flours is warranted. This study explored the effect of tempering conditions on intermediate wheatgrass flour properties, namely composition, color, solvent retention capacity, starch damage, and polyphenol oxidase activity. Changes in flour attributes were evaluated via a 3 × 3 × 2 factorial design, with factors targeting moisture (comparing un-tempered controls to samples of 12% and 14% target moisture), time (4, 8, and 24 h), and temperature (30 and 45 °C). All investigated parameters were significantly affected by target moisture; however, samples tempered to 12% moisture showed few differences to those tempered to 14%. Similarly, neither tempering time nor temperature exerted pronounced effects on most flour properties, indicating water uptake was fast and not dependent on temperature within the investigated range. Lactic acid retention capacity significantly correlated with ash (*r* = −0.739, *p* < 0.01), insoluble dietary fiber (*r* = −0.746, *p* < 0.01), polyphenol oxidase activity (*r* = −0.710, *p* < 0.01), starch content (*r* = 0.841, *p* < 0.01), and starch damage (*r* = 0.842, *p* < 0.01), but not with protein (*r* = 0.357, *p* > 0.05). In general, tempering resulted in flour with less bran contamination but only minor losses in protein.

## 1. Introduction

Consumers are increasingly interested in sustainably produced food ingredients, and alternative agricultural models have emerged to emphasize factors such as diversity and environmental concerns [1]. Perennial grains have the potential to become serious contenders in the cereal market place, provided that their quality matches the requirements of manufacturers as well as expectations of consumers [2]. The motivation for their use stems from their efficient use of water, fertilizers, and soil nutrients because of their extended root systems [3]. For instance, it has been shown that cultivation of the perennial grain intermediate wheatgrass (*Thinopyrum intermedium*, IWG) dramatically lowers environmental strains such as nitrate leaching [4].

Aside from sustainability aspects, the nutritional profile of IWG is one of its advantages for food use, due to protein and dietary fiber contents that surpass those of many other cereal grains, including wheat [5,6,7]. However, the protein makeup of IWG differs from wheat, specifically by being deficient in high-molecular weight glutenins [6,7], with deleterious effects on dough elasticity and gas holding properties [6,8]. The short domestication history of this perennial grain is reflected in a rather narrow endosperm, which, however, has been successfully increased by breeding efforts. Even though IWG kernel width is still significantly lower than for wheat [8], refinement of newer breeding materials is possible, which has been shown to retain most of the protein, while reducing especially the insoluble fiber portion [8]. In order to explore additional uses for IWG as a standalone food ingredient, processing conditions to better separate the endosperm from the bran fraction need to be investigated. Many cereal applications involve the use of flour, and, thus, milling is one of the most important processes to improve end-use characteristics of cereals. One strategy for doing so is by altering the moisture contents of kernels prior to milling. This tempering operation can increase milling efficiency [9] and affect parameters relevant for final application of the resulting flour. As there are currently no tempering conditions established for IWG, this study explored the effects of kernel moisture, tempering temperature, and tempering time on chemical characteristics of IWG. Specifically, IWG kernels were subjected to different tempering conditions in a 3 × 3 × 2 factorial design. Kernels were incubated at either 30 or 45 °C for 4, 8, or 24 h. Nontempered kernels were compared to those where water had been added to achieve either 12% or 14% target moisture. Flour samples were analyzed for composition (ash, protein, starch, and dietary fiber), polyphenol oxidase (PPO) activity, color, solvent retention capacity (SRC), and starch damage. Initial work on IWG incorporation into baked goods utilized it in whole-grain form [6]. In a previous study, properties of doughs made of completely, partially, and unrefined IWG were compared, as well as the influence of addition of commercial dough conditioners on these properties in relation to refinement [8]. It was noticed that dough conditioners had more pronounced effects on dough properties in completely refined IWG [8]. In addition, crumb characteristics of breads were improved when ascorbic acid was part of the recipes, but only when flours had been completely refined [10]. However, the flours used in these experiments had not been tempered before milling; therefore, additional refinement due to better bran separation may have yielded further enhanced properties. Thus, this study is part of a wider reaching effort to improve IWG functionality for baking applications to facilitate its incorporation into a wide array of products with desirable properties. The overall objective of this wider study is to optimize the tempering conditions of IWG for end use applications. For this study, we focused on the effects of tempering on the chemical composition, color, solvent retention capacity, starch damage, and polyphenol oxidase activity.

## 2. Materials and Methods

IWG variety MN1503 was grown and harvested in Becker, Minnesota, USA. All chemicals used were of reagent grade or higher. Tempering as well as chemical analyses were carried out according to American Association of Cereal Scientists International (AACCI) official methods [11] unless otherwise noted.

Samples were tempered in duplicates at 4, 8, or 24 h at either 30 or 45 °C. Kernels were either used at intrinsic moisture (i.e., no water added) as controls or tempered to 12% ± 0.5% or 14% ± 0.5% target moisture. Tempering was performed by weighing 50 g of IWG kernels into air-sealed glass jars, to which the required amount of tempering water was added, hand mixed using a spatula for 60 s, shaken for another 60 s to ensure equal distribution of water, and tempered in an incubator at each condition with fixed relative humidity of 50%. The amount of tempering water to be added to each tempering condition was calculated as per AACCI method 26-10.02.

At the end of each tempering condition, duplicates of 1 g of kernels were removed for whole kernel moisture analysis via AACCI method 44-16.01, and remaining kernels were milled using a Brabender Quadrumat Junior mill (Model12-02-000, C.W. Brabender Instruments, Hackensack, NJ, USA). To avoid flour losses, bran was sifted manually using a 250 μm sieve plate. Flour with particle sizes >250 μm was collected as bran and flour with particle sizes <250 μm as endosperm. Moisture contents of the freshly milled flour were determined on an Ohaus MB45 (Parsippany, NJ, USA).

Flours were analyzed for ash (AACCI method 08-01.01), protein (AACCI method 46-30.01) on a Leco FP 828 (Leco 165 Corporation, St. Joseph, MI, USA), color (AACCI method 14.22.01) Chroma Meter CR-221 (Minolta Camera Co., Osaka, Japan), dietary fiber (AACCI method 32-07.01), SRC (AACCI method 56-11.02), total starch (AACCI method 76-13.01), and starch damage (AACCI method 76-31.01). For dietary fiber, total and damaged starch, test kits from Megazyme (Wicklow, Ireland) were used. The activity of polyphenol oxidases (PPOs) were determined in flour based on a previously reported procedure [12]. Briefly, 200 mg of flour were used to assess oxidation of 3,4-dihydroxy-l-phenylalanine by measuring A_475_ of its conversion product on a UV1800 (Shimadzu, Columbia, MD). The substrate was dissolved in 50 mM 3-morpholinopropane-1-sulfonic acid buffer adjusted to pH 6.5 with HCl, containing 0.02% Tween-20. Samples were shaken for 1 h on a Fisher Pulsing Vortex mixer operated at a speed setting of 2500 (Fisher Scientific, Waltham, MA, USA). Enzyme activity is reported as absorbance change ΔA_475_ min^−1^ g^−1^ flour dry basis, as outlined previously [12].

Three-way analysis of variance (ANOVA) was performed in R (version 3.1.0, R Core Team, 2013) using target moisture, tempering time, and temperature as factors. All analyses were carried out at least in duplicate and are reported as means on a dry basis ± standard deviation. Differences among means were considered significant if *p* < 0.05 and were compared within each factor. Only significant differences and correlations are reported. One-way ANOVAs followed by least significance difference (LSD) tests were run in R to evaluate differences according to target moisture and tempering time. Paired *t*-tests were conducted in Microsoft Excel^®^ (2013) to evaluate differences according to tempering temperature. Pearson-type correlation coefficients and principal component analyses were done in R to summarize relationships among the data.

## 3. Results and Discussion

### 3.1. Flour and Kernel Moisture Uptake During Tempering

Target moisture exerted the greatest effect on kernel as well as flour moisture, in comparison to temperature and time (Table 1). For all tempering conditions, nontempered samples had significantly lower flour and kernel moisture than samples tempered to 12% or 14% moisture. The fact that these differences were already significant after 4 h indicated rapid water uptake. The observed differences in the moisture contents of the nontempered samples shown in Table 1 were due to the fact that kernel moisture was determined prior to each tempering experiment, even though kernels were from the same batch. This was necessary because not all tempering experiments could be performed on the same day. Incubation time only affected kernel moisture values obtained for nontempered samples at 30 °C and IWG tempered to 12% target moisture at 45 °C. The length of tempering did not cause significant differences in flour moisture of samples tempered to either 12% or 14% target moisture. In wheat, tempering times are often optimized for cultivars, and lengths of 1–24 h are common [13]. However, water uptake is not uniform across grain tissue layers because of the differences in water permeability [13]. Ultimately, the goal of tempering is to facilitate separation of endosperm and bran by plasticizing the former while concomitantly toughening the latter.

For samples with 14% target moisture, more variability between replicate tempering treatments was observed, and not all samples reached the intended 14% level, which resulted in the differences between 12% and 14% target moisture as not being significant. Regardless of tempering conditions, flour moisture was lower than the corresponding kernel moisture. This could be due to the loss of moisture from the flour during the milling process. As seen in Table 1, target moisture showed significant differences in the flour moisture values recorded for all samples, excluding tempering conditions 30 °C 24 h 12% and 30 °C 24 h 14% wherein no significant differences were observed. Tempering temperature did not significantly affect kernel moisture except for samples tempered for 8 h to 12% or 14% target moisture. Moreover, temperature did not significantly affect flour moisture of any samples tempered to 12% and 14% target moisture.

### 3.2. Color

Similar to flour and kernel moisture, the target moisture was the most impactful factor on color (Table 2). All samples tempered to 12% or 14% target moisture had significantly lower *a* values, and most also had significantly higher *L* and *b* values. Thus, tempering resulted in flours that were lighter, less reddish/brown, but more yellow than nontempered controls. These results indicate successive changes in flour refinement with increased target moisture, as darker, more reddish/browner but less yellow samples contain more bran. No significant differences according to tempering length or temperature were detected.

### 3.3. Flour Composition

#### 3.3.1. Ash, Insoluble Dietary Fiber, and Protein

Cereal bran in general, and IWG bran in particular, is richer in ash and dietary fiber (particularly insoluble dietary fiber) than refined flour [8]. Thus, higher ash values are often taken as a proxy for bran contamination of endosperm [9]. Target moisture significantly affected ash and insoluble dietary fiber in a similar way as observed for color (Table 2): all nontempered control flours contained significantly more ash and insoluble fiber than samples tempered to either 12% or 14% target moisture. In contrast, refinement did not always lead to protein loss when considering values on a dry basis. It has been shown before that IWG surpasses many other cereals, including wheat, in protein content [5,6,7,8], and this characteristic is maintained over the refinement process. From a nutritional perspective, a target moisture of 12% may be more desirable than 14% since it often results in more insoluble fiber, while not differing in ash contents to those samples. However, future studies would need to evaluate if dough rheological properties are different between such samples. It was previously observed that dough viscoelasticity of IWG is detrimentally affected by the presence of bran [8]. The effects of tempering time and temperature on the compositional attributes reported in Table 3 were minor compared to target moisture. However, tempering for 8 h at 45 °C resulted in lower ash contents than tempering for 4 or 24 h.

Ash and insoluble dietary fiber contents displayed significant, negative correlations with *L* (−0.849 and −0.909, respectively; *p* < 0.01 for both) and *b* values (*r* = −0.824 and −0.887, respectively, *p* < 0.01 for both) and significant and positive correlations with *a* values (*r* = 0.898 and 0.934, respectively, *p* < 0.01 for both). The negative relationship between lightness and ash contents has been reported numerous times for other cereal flours, notably wheat [14] and rye milling fractions [15]. However, for rye it was observed that lighter flours were not only less red, but also less yellow, because of the lower brownness [15]. This discrepancy to IWG may be related to differences in carotenoid contents since these would contribute to yellowness more than to redness. The contents of zeaxanthin, and especially lutein, in IWG exceeded ranges reported for many other cereals such as wheat [7] and are the reason for high *b* values (Table 2) in comparison to flours from, for example, wheat [16]. In contrast to the relation between color values and ash, correlations between *L*, *a*, and *b* to protein contents were not significant. Protein did, however, exhibit a positive correlation with ash (*r* = 0.843, *p* < 0.01) but not with insoluble dietary fiber.

#### 3.3.2. Starch and Damaged Starch

**Figure 1:**
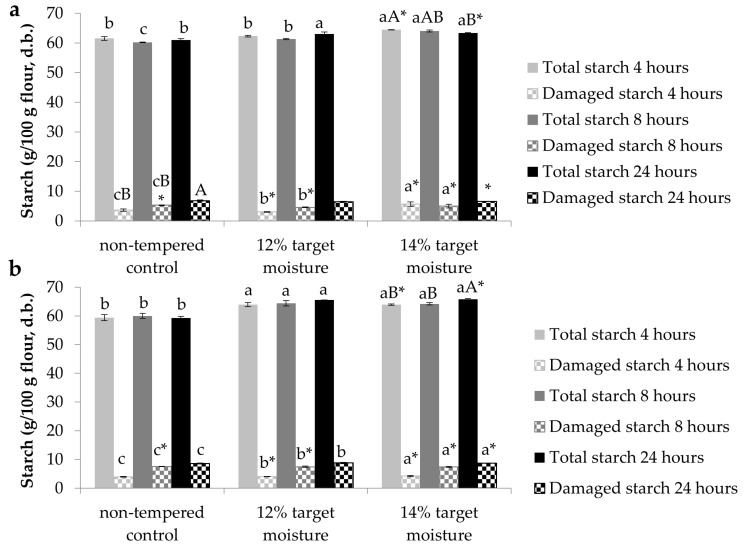
Starch and damaged starch contents in (**a**) samples incubated at 30 °C and (**b**) samples incubated at 45 °C. Lowercase letters reflect differences according to target moisture, uppercase letters illustrate differences due to incubation time, and asterisks represent differences due to incubation temperature.

The ash and insoluble fiber reductions achieved via tempering were reflected in significantly higher starch contents with higher target moisture (Figure 1a,b). This was the case at both tempering temperatures and for all incubation times except for 4 h at 30 °C, where the difference between nontempered control flour and 12% target moisture was not significant. As observed for color and the other evaluated constituents (Table 3), few differences between tempering length and temperature were significant. The starch values observed here were higher than in previous studies [5,6,7], which, however, had been measured on whole-grain IWG from different breeding stages.

The increases in starch contents coincided with increases in starch damage, which ranged from 3.04% ± 0.10% to 8.78% ± 0.24% (Figure 1a,b). While the values at the low range were in line with previous reports [17], and comparable to some soft wheat varieties [18,19] or rye [15], the values at the upper range were closer to medium-hard [20] or even hard wheat varieties [21]. Different from the compositional attributes shown in Table 3, tempering temperature caused more differences among samples. Except for samples incubated for 24 h at 12% target moisture, all tempered samples had significantly higher starch damage when incubated at 45 °C. Starch and starch damage exhibited significant, positive correlations with *L* (*r* = 0.760 and 0.692, respectively; *p* < 0.01 for both) and *b* (*r* = 0.807 and 0.809, respectively; *p* < 0.01 for both) and significant, negative correlations with *a* (*r* = −0.817 and −0.767, respectively, *p* < 0.01 for both). These relationships essentially indicate that all these parameters assess the extent of IWG refinement. While tempering may reduce the extent of starch damage by softening the kernels, some studies have found more starch damage in refined than whole wheat flours and attributed this to starch dilution by bran in whole wheat [19]. At high extents, starch damage can negatively influence product properties (e.g., lower functionality and sensory properties [22]), but below a certain threshold it may also be beneficial for product quality [23].

### 3.4. Polyphenol Oxidase (PPO) Activity

**Figure 2:**
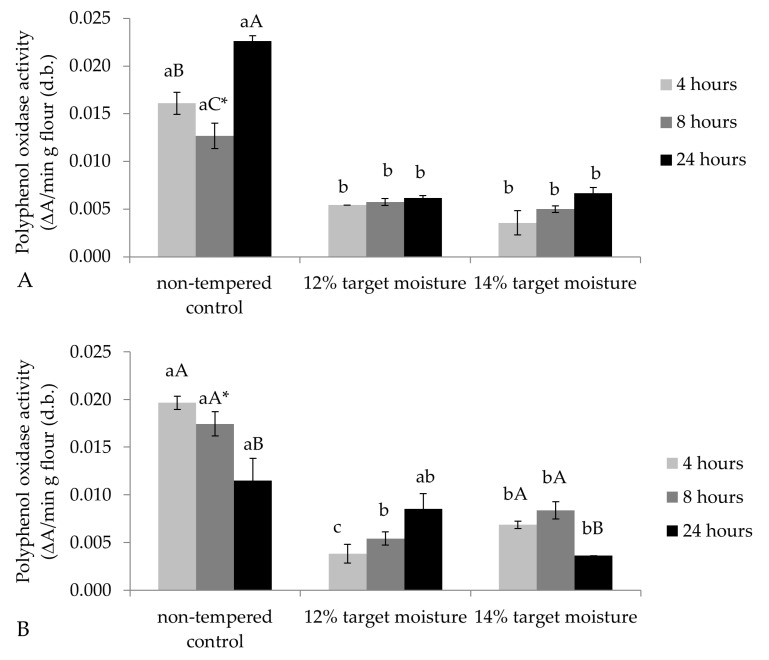
Polyphenol oxidase activities in (**A**) samples incubated at 30 °C and (**B**) samples incubated at 45 °C. Lowercase letters reflect differences according to target moisture, uppercase letters illustrate differences due to incubation time, and asterisks represent differences due to incubation temperature.

Aside from ash contents, PPO activity is often cited as another indicator of bran contamination [9]. PPO activity can lead to discoloration and specks in products such as noodles. The values observed in our study were similar to those reported for PPO activity in wheat flour [12] and follow the same pattern as color, ash, and insoluble fiber data: nontempered controls had significantly higher PPO activity than samples of 12% or 14% target moisture, while incubation time and temperature had comparably minor effects (Figure 2a,b). Thus, similar to previous studies [9], PPO activity reflected refinement increases brought about by tempering and, consequently, was highly, positively correlated with *a* values (*r* = 0.895, *p* < 0.01), ash (*r* = 0.843, *p* < 0.01), and insoluble dietary fiber (*r* = 0.921, *p* < 0.01) and negatively correlated with starch (*r* = −0.727, *p* < 0.01), *L* (*r* = −0.890, *p* < 0.01), and *b* values (*r* = −0.808, *p* < 0.01). However, there was no significant correlation between PPO activity and protein contents (Table S1) because bran reduction did not decrease protein contents to a similar extent than it reduced PPO activity (Table 3). The low PPO activity in combination with high *b* values of the tempered IWG flours makes them attractive candidates for product applications such as noodles.

### 3.5. Solvent Retention Capacity

Flour swelling in lactic acid is related to gluten formation and is, thus, related to protein quality [24]. For samples tempered at 30 °C for 4 or 24 h, as well as samples tempered at 45 °C for 4 or 8 h, nontempered controls had significantly lower lactic acid SRCs than samples of 12% or 14% target moisture (Table 4). While the lactic acid SRC values were lower than for hard wheat, they were higher than previously reported for refined IWG [17]. This indicates that when the extent of refinement is increased, IWG flours develop stronger protein networks, which would be beneficial for the manufacture of products that require gas holding properties. The fact that several tempering conditions resulted in flour with significantly increased lactic acid SRC may be related to lower bran contents in these flours and, thus, less interference with protein network formation. In a previous study on IWG, bran addition to refined IWG resulted in changes in protein secondary structure distributions, with refined IWG having significantly fewer β-sheets, but more β-turns, than whole IWG [8]. Such changes reflect water redistribution, due to bran constituents, and a less hydrated protein network in whole IWG according to the loop and train model [25]. Thus, a higher lactic acid SRC may be indicative of better viscoelastic properties, which need to be evaluated in future studies. Lactic acid SRC was positively correlated with *L* (*r* = 0.805, *p* < 0.01), *b* (*r* = 0.831, *p* < 0.01), starch content (*r* = 0.841, *p* < 0.01), and also starch damage (*r* = 0.842, *p* < 0.01); accordingly, negative correlations were present with *a* (*r* = −0.837, *p* < 0.01), ash (*r* = −0.739, *p* < 0.01), insoluble dietary fiber (*r* = −0.746, *p* < 0.01), and PPO activity (*r* = −0.710, *p* < 0.01). Several previous studies have shown correlations between lactic acid SRC and protein or gluten content [21]; however, correlations with protein content were not always present when different wheat varieties were compared to each other [26]. Since, overall, gluten network formation, and consequently baking quality, is affected by the interplay among flour polymers, most importantly gluten-forming proteins, damaged starch, and arabinoxylans [24]. There are also reports that indicates that lactic acid SRC correlations with starch content may not always be significant and can be positive [27,28]. The positive correlations of lactic acid SRC with *L*, *b*, and starch as well as the negative correlations with *a*, ash, and insoluble dietary fiber are likely a reflection of the increasing degree of refinement brought upon by tempering. The lack of a significant correlation with protein likely is due to protein contents remaining relatively constant after tempering; thus, the increase in starch came at the expense of insoluble fiber.

Swelling of flour in sodium carbonate solution is the result of starch damage [24]. However, sodium carbonate SRC followed a similar, yet less pronounced, pattern as all parameters that increased with bran contamination. In particular, nontempered samples incubated at 45 °C had significantly higher sodium carbonate SRC values than samples incubated for the same time to either 12% or 14% target moisture (Table 4). These results align with reports of tempering softening kernels and facilitating the separation of endosperm from bran layers without rupturing starch granules [29]. However, they were not significantly correlated to the results of the starch damage assay, which measures starch digestion via amylase. In addition, microscopy of granules would be informative. Sodium carbonate SRC exhibited significant, but moderate, correlations with *L* (*r* = −0.527, *p* < 0.05), *a* (*r* = 0.566, *p* < 0.05), insoluble fiber (*r* = 0.605, *p* < 0.01), starch content (*r* = −0.557, *p* < 0.05), and PPO activity (*r* = 0.564, *p* < 0.05).

Sucrose SRC is mediated by arabinoxylan swelling, which is an important constituent of IWG dietary fiber [30]. These results showed correlations of similar, moderate magnitudes than sodium carbonate SRC, but relationships were inverted: sucrose SRC had a significant, positive correlation with *b* values (*r* = 0.485, *p* < 0.05) and starch damage (*r* = 0.614, *p* < 0.01) and negative correlations with *a* (*r* = −0.487, *p* < 0.05) and protein (*r* = −0.598, *p* < 0.01).

SRC in water is affected by all flour polymers [24] but was a less informative parameter for our sample set. Only correlations with protein (*r* = −0.627, *p* < 0.01) and starch damage (*r* = 0.500, *p* < 0.05) were significant.

Previously, refined but nontempered IWG was reported to have lactic acid SRC, sodium carbonate SRC, sucrose SRC, and water SRC of 78.7, 98.1, 107.2, and 73.3, respectively [17]. Thus, the additional refinement achieved via tempering resulted in increases in SRC values for all solvents (Table 4).

### 3.6. Principal Component Analysis

**Figure 3:**
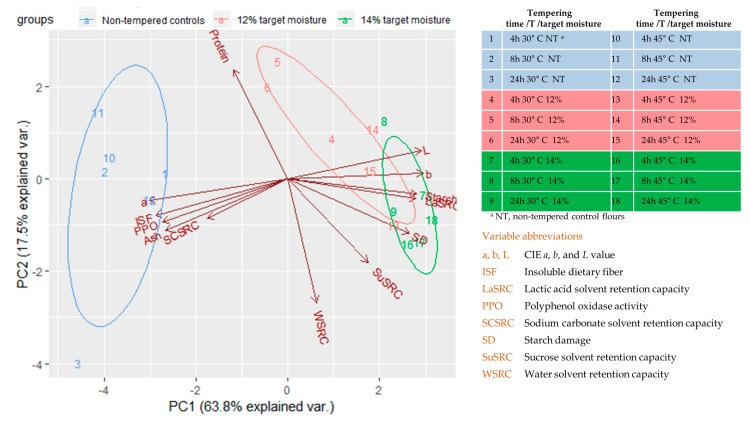
Principal component (PC) analysis of intermediate wheatgrass flours obtained via various tempering conditions.

The characteristics of the flour samples were summarized via a principal component analysis where the first two principal components accounted for >80% of the variability in the data set (Figure 3). Nontempered controls were distinctly separated from samples of 12% and 14% target moisture via principal component 1, which was negatively correlated with *a*, insoluble dietary fiber, ash, and PPO activity, while *L*, *b*, starch contents, and lactc acid SRC had high, positive correlations. Principal component 2 was positively correlated with protein contents and negatively correlated with water SRC. However, potentially because of the low variability in these properties, sample groups could not be effectively separated via principal component 2. As a result, differentiation between samples of 12% to those of 14% target moisture could not be achieved. In addition, samples could also not be separated based on incubation temperature or time (Figure S1a,b).

## 4. Conclusions

This study has shown that tempered IWG samples have significantly different flour properties compared to nontempered controls. Rapid moisture uptake resulted in few significant differences in kernel or flour moisture among samples, which likely affected all other evaluated parameters. Tempering produced lighter, less brown, more yellow flour with more starch as well as more damaged starch, but it reduced polyphenol oxidase activity. To better establish the tempering parameters for IWG, future work will assess the functional attributes of tempered and refined IWG flours.

## Figures and Tables

**Table 1:** Intermediate wheatgrass kernel and flour moisture as affected by tempering conditions.

30 °C	Incubation Time					
	4 h		8 h		24 h	
Target Moisture (%)	Kernel	Flour	Kernel	Flour	Kernel	Flour
NT ^1^	8.85 ± 0.05 ^c^	8.94 ± 0.04 ^C^*	9.00 ± 0.05 ^c^	9.22 ± 0.15 ^C^	9.22 ± 0.02 ^c^	8.47 ± 0.02 ^B^
12	11.88 ± 0.27 ^b^	10.68 ± 0.07 ^B^	12.10 ± 0.06 ^b^*	10.09 ± 0.07 ^B^	11.51 ± 0.49 ^b^	10.7 ± 0.45 ^A^
14	13.93 ± 0.35 ^a^	12.06 ± 0.20 ^A^	14.07 ± 0.01 ^a^*	12.02 ± 0.23 ^A^	13.45 ± 0.48 ^a^	11.44 ± 0.20 ^A^
45 °C	4 h		8 h		24 h	
Target Moisture (%)	Kernel	Flour	Kernel	Flour	Kernel	Flour
NT	8.72 ± 0.03 ^b^	8.59 ± 0.02 ^C^*	9.02 ± 0.25 ^c^	9.09 ± 0.14 ^C^	9.09 ± 0.03 ^c^	8.26 ± 0.10 ^C^
12	12.62 ± 0.06 ^a^	11.21 ± 0.11 ^B^	12.01 ± 0.06 ^b^*	11.05 ± 0.06 ^B^	11.76 ± 0.05 ^b^	11.17 ± 0.03 ^B^
14	13.42 ± 0.54 ^a^	12.32 ± 0.09 ^A^	13.61 ± 0.01 ^a^*	12.06 ± 0.14 ^A^	13.45 ± 0.01 ^a^	12.24 ± 0.03 ^A^

^1^ NT, nontempered control flours. Different letters represent significant (*p* < 0.05) differences among samples incubated at the same temperature for the same length of time, but to different target moistures. Lowercase letters denote differences for kernels; uppercase letters indicate differences among flour samples. Asterisks represent differences due to incubation temperature.

**Table 2:** Color of intermediate wheatgrass flour subjected to different tempering conditions.

*L*	Incubation Time/Temperature					
	4 h		8 h		24 h	
Target Moisture (%)	30 °C	45 °C	30 °C	45 °C	30 °C	45 °C
NT	85.90 ± 0.63 ^b^	85.35 ± 0.86 ^b^	85.75 ± 0.43 ^b^	86.25 ± 0.31	84.70 ± 0.28 ^b^	86.11 ± 0.59 ^b^
12	88.34 ± 0.11 ^a^	87.89 ± 0.22 ^a^	88.09 ± 0.17 ^a^	88.03 ± 0.26	86.84 ± 1.25 ^ab^	87.76 ± 0.35 ^a^
14	88.57 ± 0.25 ^a^	88.25 ± 0.14 ^a^	88.72 ± 0.01 ^a^	88.51 ± 0.22	88.98 ± 0.04 ^a^	88.14 ± 0.07 ^a^
*a*						
NT	−6.19 ± 0.06 ^a^	−5.99 ± 0.00 ^a^	−5.86 ± 0.04 ^a^	−6.07 ± 0.16 ^a^	−5.52 ± 0.35 ^a^	−6.10 ± 0.11 ^a^
12	−6.93 ± 0.05 ^b^	−7.05 ± 0.07 ^b^	−6.83 ± 0.07 ^b^	−6.93 ± 0.08 ^b^	−6.58 ± 0.19 ^b^	−6.78 ± 0.07 ^b^
14	−7.15 ± 0.11 ^b^	−7.14 ± 0.02 ^b^	−7.06 ± 0.11 ^b^	−7.23 ± 0.06 ^b^	−7.08 ± 0.13 ^b^	−7.09 ± 0.05 ^c^
*b*						
NT	19.24 ± 0.69 ^b^	18.95 ± 0.11 ^c^	18.90 ± 0.29 ^c^	19.33 ± 0.23 ^c^	18.27 ± 0.65	19.24 ± 0.56 ^b^
12	20.32 ± 0.06 ^ab^	20.56 ± 0.03 ^b^	20.35 ± 0.31 ^b^	20.69 ± 0.48 ^b^	19.58 ± 1.14	20.51 ± 0.34 ^ab^
14	21.32 ± 0.33 ^a^	21.34 ± 0.36 ^a^	21.40 ± 0.07 ^a^	21.84 ±0.31 ^a^	21.32 ± 0.13	21.29 ± 0.35 ^a^

^1^ NT, nontempered control flours. Different lowercase letters represent significant (*p* < 0.05) differences among samples incubated at the same temperature and for the same length, but tempered to different target moistures (i.e., across columns). Target moisture did not significantly affect *L* values of samples incubated at 45 °C for 8 h and *b* values of samples incubated at 30 °C for 24 h.

**Table 3:** Ash, insoluble dietary fiber, and protein contents of intermediate wheatgrass flour (g/100 g, dry basis) as affected by tempering conditions.

		Incubation Time/Temperature					
	Target Moisture (%)	4 h		8 h		24 h	
		30 °C	45 °C	30 °C	45 °C	30 °C	45 °C
Ash							
	NT ^1^	0.82 ± 0.00 ^aB^	0.80 ± 0.04 ^a^	0.90 ± 0.01 ^aB^	0.79 ± 0.01 ^a^	1.02 ± 0.05 ^aA^	0.77 ± 0.03 ^a^
	12	0.69 ± 0.01 ^b^	0.66 ± 0.01 ^bA^	0.70 ± 0.04 ^b^	0.59 ± 0.01 ^bB^	0.71 ± 0.02 ^b^*	0.63 ± 0.02 ^bA^*
	14	0.68 ± 0.04 ^b^	0.67 ± 0.02 ^b^	0.71 ± 0.03 ^b^*	0.65 ± 0.03 ^b^*	0.70 ± 0.03 ^b^	0.64 ± 0.01 ^b^
Insoluble dietary fiber							
	NT	5.81 ± 0.39 ^a^	7.45 ± 0.12 ^aA^	6.83 ± 0.55 ^a^	6.93 ± 0.24 ^aA^	8.50 ± 1.18 ^a^	6.46 ± 0.31 ^aB^
	12	4.39 ± 0.51 ^b^	3.90 ± 0.22 ^b^	4.07 ± 0.50 ^b^	4.31 ± 0.41 ^b^	4.55 ± 0.43 ^b^	3.65 ± 0.17 ^b^
	14	2.37 ± 0.02 ^cB^	3.97 ± 0.28 ^bA^	2.79 ± 0.96 ^bB^*	4.00 ± 0.00 ^bA^*	4.69 ± 0.12 ^bA^	3.22 ± 0.18 ^bB^
Protein							
	NT	19.25 ± 0.10	19.45 ± 0.06 ^aAB^	19.35 ± 0.14	19.57 ± 0.08a ^A^	19.01 ± 0.06 ^b^	19.28 ± 0.03 ^B^
	12	19.34 ± 0.19	19.19 ± 0.02 ^b^	19.39 ± 0.05	19.21 ± 0.03 ^b^	19.37 ± 0.04 ^a^	19.26 ± 0.02
	14	19.04 ± 0.05	19.11 ± 0.10 ^b^	19.48 ± 0.29	18.99 ± 0.06 ^c^	19.16 ± 0.06 ^b^	19.27 ± 0.09

^1^ NT, nontempered control flours. Different lowercase letters signify differences among samples incubated according to target moisture (i.e., across columns). Different uppercase letters denote differences among samples according to tempering time, and asterisks represent differences due to incubation temperature.

**Table 4:** Solvent retention capacity (SRC) of tempered intermediate wheatgrass flour.

Lactic Acid SRC	Incubation Time/Temperature					
	4 h		8 h		24 h	
Target Moisture (%)	30 °C	45 °C	30 °C	45 °C	30 °C	45 °C
NT ^1^	93.7 ± 1.3 ^bA^	96.9 ± 1.7 ^bA^	88.9 ± 0.0 ^B^	88.1 ± 0.9 ^bB^	92.8 ± 0.8 ^bA^	94.0 ± 0.8 ^A^
12	100.8 ± 1.1 ^aA^*	107.2 ± 0.9 ^a^*	95.5 ± 0.4 ^B^	105.5 ± 0.9 ^a^	97.8 ± 0.8 ^bB^*	105.4 ± 0.7 ^*^
14	103.2 ± 0.2 ^a^	102.8 ± 1.4 ^a^	106.5 ± 8.0	104.4 ± 1.3 ^a^	112.2 ± 6.1 ^a^	109.6 ± 7.5
Sodium carbonate SRC						
NT	111.0 ± 0.2 *	115.9 ± 1.2 ^aB^*	112.6 ± 3.9 *	129.0 ± 3.9 ^aA^*	121.8 ± 4.7 ^a^*	132.7 ± 1.9 ^aA^*
12	109.2 ± 1.5 *	113.6 ± 0.3 ^ab^*	108.1 ± 2.1 *	110.9 ± 3.1 ^b^*	105.2 ± 2.8 ^b^	112.1 ± 0.6 ^b^
14	108.4 ± 0.8	110.1 ± 1.5 ^b^	111.5 ± 1.4	113.7 ± 0.2 ^b^	110.5 ± 3.2 ^ab^*	114.2 ± 1.4 ^b^*
Sucrose SRC						
NT	131.1 ± 1.4 ^b^	130.4 ± 4.5 ^b^	123.3 ± 0.5 ^b^	126.7 ± 3.7	142.5 ± 14.2	143.8 ± 17.6
12	149.3 ± 3.2 ^a^	150.2 ± 0.1 ^a^	124.9 ± 2.2 ^b^	132.2 ± 8.0	128.8 ± 10.9	139.9 ± 3.4
14	147.0 ± 6.2 ^a^	147.4 ± 1.3 ^a^	139.6 ± 3.9 ^a^	143.7 ± 0.9	141.3 ± 3.7	140.9 ± 3.2
Water SRC						
NT	89.1 ± 0.4	88.4 ± 1.6 ^b^	91.6 ± 1.5 ^a^	86.3 ± 0.4 ^b^	94.5 ± 3.6 *	87.9 ± 1.0 ^b^*
12	87.4 ± 1.5 *	91.9 ± 1.0 ^aA^*	86.3 ± 0.8 ^b^	86.8 ± 1.7 ^bB^	86.0 ± 3.2	89.1 ± 0.0 ^bAB^
14	89.6 ± 0.9 *	93.1 ± 0.0 ^aAB^*	89.1 ± 0.9 ^ab^*	91.4 ± 0.5 ^aB^*	92.1 ± 5.1	94.0 ± 0.8 ^aA^

^1^ NT, nontempered control flours. Lowercase letters show differences according to target moisture, uppercase letters represent differences due to incubation time, and asterisks indicate differences due to incubation temperature.

## Supplementary Materials

The following are available online at https://www.mdpi.com/2304-8158/8/8/337/s1, Figure S1: Principal component (PC) analysis of intermediate wheatgrass flour obtained after different tempering treatments. Ellipses represent sample categorization via (a) temperature; (b) incubation time, Table S1: Pearson correlation coefficients among intermediate wheatgrass flour parameters.
