# Supplementary material for: Tempering Improves Flour Properties of Refined Intermediate Wheatgrass (Thinopyrum intermedium)
Source: Foods. 2019 Aug 10;8(8):337. doi: 10.3390/foods8080337 (PMC6723072; doi:10.3390/foods8080337)
Supplement: Supplementary file 1 [file foods-08-00337-s001.pdf]

Table S1. Pearson correlation coefficients among intermediate wheatgrass flour parameters. Values in bold followed by one or two asterisks reflect significant correlations at $p < 0.05$ and $p < 0.01$, respectively.

	**LaSRC**	**SCSRC**	**SuSRC**	**WSRC**	***L***	***a***	***b***	**SD**	**PPO**	**Starch**	**Protein**	**Ash**	**ISF**
LaSRC		-0.456	**0.580***	0.320	**0.805****	**-0.837****	**0.831****	**0.842****	**-0.710****	**0.841****	-0.357	**-0.739****	**-0.746****
SCSRC			0.005	0.005	**0.527***	**0.566***	-0.458	-0.315	**0.564***	**-0.557***	-0.330	-0.327	**0.605****
SuSRC				**0.477***	0.446	**-0.487***	**0.485***	**0.614****	-0.367	**0.466**	**-0.598****	0.358	-0.395
WSRC					0.021	-0.061	0.183	**0.500***	-0.627	**0.301**	**-0.627****	0.219	0.023
L						-0.972	0.953	**0.692****	**-0.890****	**0.7604****	0.194	**-0.849****	**-0.909****
a								**-0.767****	**0.895****	**-0.817****	0.260	**0.898****	**0.934****
b								**0.809****	**-0.808****	**0.807****	-0.312	**-0.824****	**-0.887****
SD									**-0.584***	**0.872****	**-0.564***	**-0.664***	**-0.665***
PPO										**-0.727****	0.123	**0.843****	**0.921****
Starch											-0.412	**-0.733****	**-0.837****
Protein												**0.843****	0.165
Ash													**0.853****

LaSRC: lactic acid solvent retention capacity; SCSRC: sodium carbonate solvent retention capacity; SuSRC: sucrose solvent retention capacity; WSRC: water solvent retention capacity; L: CIE *L* value; a: CIE *a* value; b: CIE *b* value; SD: Starch Damage; PPO: Polyphenoloxidase activity; TS: Total Starch; ISF: Insoluble Fiber.

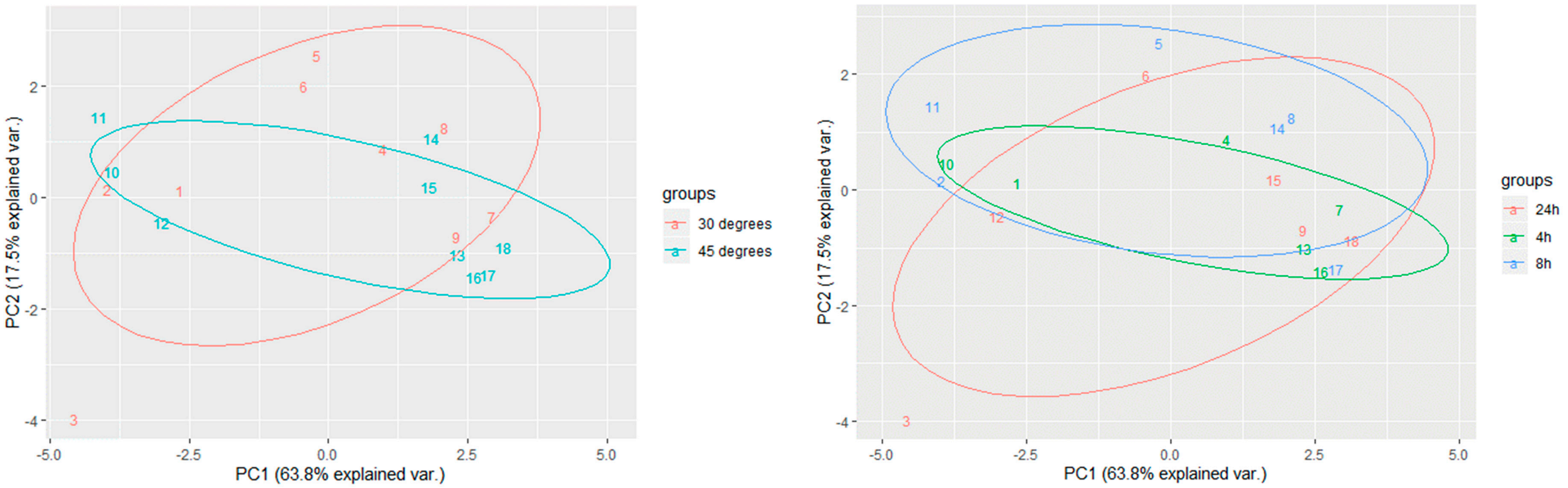

Figure S1. Principal component (PC) analysis of intermediate wheatgrass flour obtained after different tempering treatments. Ellipses represent sample categorization via (a) temperature; (b) incubation time.
